# Study of Serum Leptin in Well-differentiated Thyroid Carcinoma: Correlation with Patient and Tumor Characteristics

**DOI:** 10.1007/s00268-014-2634-8

**Published:** 2014-05-28

**Authors:** Rania Abdel Rehem, Waleed Abo Elwafa, Reham Abo Elwafa, Tarek Ezzat Abdel-Aziz

**Affiliations:** 1 Internal Medicine Department, Endocrinology, Faculty of Medicine, Alexandria University, Alexandria, Egypt; 2 Department of General Surgery, Endocrine Unit, Faculty of Medicine, Alexandria University, Alexandria, Egypt; 3 Department of Clinical Pathology, Faculty of Medicine, Alexandria University, Alexandria, Egypt; 4 General and Endocrine Surgery Unit, University College London Hospital, 235 Euston Road, London, NW1 2BU UK; 5 Division of Surgery and Interventional Science, University College London, London, UK

## Abstract

**Background:**

There is a proven relationship between obesity and several cancers including breast, endometrium, colorectal, and esophagus. With the increasing incidence of both obesity and thyroid cancer, we designed the present study to investigate a causal relationship between leptin, which is one of the well known adipokines, and well-differentiated thyroid cancer (WDTC).

**Methods:**

Serum leptin levels were measured in 30 patients with WDTC and compared to 30 healthy control subjects before and 1 month after surgery. Other parameters studied included age, sex, body mass index, menopausal status in women, lymph node status, tumor size, and disease multifocality.

**Results:**

There were no differences between the two groups regarding age and sex. Preoperative leptin levels were higher in the WDTC patients when compared to the control patients [19.25 (1.50–109.60) vs 0.90 (0.50–11.80) ng/ml, *p* < 0.001, group 1 vs group 2, respectively]. A significant drop in leptin levels 1 month after surgery occurred in the WDTC group, falling from 19.25 (1.50–109.60) to 0.90 (0.60–8.90) ng/ml (*p* < 0.001). This did not occur in the control group (*p* = 0.274). Lymph node involvement, tumor size, and multifocality had no effect on leptin levels, although trends were observed (*p* = 0.48, 0.079, and 0.064), respectively.

**Conclusions:**

Serum leptin levels were significantly higher in WDTC patients when compared to control group patients, with a significant drop after surgery. Leptin may play a role in diagnosis of WDTC; however, its prognostic value is still undetermined.

## Introduction

Obesity is a worldwide major health issue with an increasing prevalence [1]. In addition to being a medical condition increasing the risk of heart disease and diabetes, obesity is associated with several cancers through a family of metabolically active adipocytokines including tumor necrosis factor (TNF)-alpha, interleukin (IL)-6, type 1 plasminogen activator inhibitor, adiponectin, and leptin [2–5]. The exact mechanisms explaining this association are still at large; however, several theories have been proposed, including hyperinsulinemia and a state of chronic low level inflammation associated with obesity, with abnormal cytokine production affecting tumor initiation and progression [6, 7]. Leptin, one of the most abundant and most investigated adipokines, was thought to have a role in regulating body weight solely by regulating body fat stores, but leptin has functions that extend beyond food intake and energy balance regulation, being also a metabolic and neuroendocrine hormone. Leptin has roles in glucose metabolism, normal sexual maturation, and reproduction, and it also has effects on the hypothalamic–pituitary–adrenal, thyroid and growth hormone axes, hematopoiesis, and the immune system [8, 9]. With respect to its diverse functions in immunity, leptin may have a major role in oxidation, which is an important risk factor in carcinogenesis. Indeed, obesity has long been recognized to be a risk factor for tumorigenesis, and accumulating evidence suggests that leptin is a potential link between obesity and cancer development. Further studies have shown it to act as a mitogenic agent promoting the proliferation and invasiveness of certain cancer cells [10].

Papillary thyroid carcinoma (PTC) is the most common neoplasm of the thyroid, accounting for 80–85 % of all thyroid cancers [11]. Increasing incidence of differentiated thyroid cancers has been observed in both men and women across all tumor sizes [12]. PTC is found in a variety of morphologic variants, like the follicular variant of papillary thyroid carcinoma (FVPTC). It is usually clinically indolent, although rarely it may present with local invasion or distant metastases, which may adversely affect survival [13]. The relation between obesity and thyroid cancer has recently been studied, showing a higher prevalence of thyroid cancer in the obese population [14]. The present study was designed to investigate the relation between serum levels of leptin and well-differentiated thyroid carcinoma (WDTC) and to evaluate its correlation with patient and tumor characteristics.

## Methods

Sixty patients were enrolled in this study and were divided into two groups. Group 1 (*n* = 30) comprised patients with WDTC diagnosed by fine needle aspiration cytology (FNAC), and group 2 (*n* = 30) who were the control patients with benign thyroid nodules. All patients included in this study were admitted to Alexandria Main University hospital between September 2011 and September 2012. An informed consent form was signed by all patients according to a protocol approved by the Ethics Committee of the hospital.

All patients were subjected to complete physical examination, standard biochemical tests, and thyroid function tests. Both FNAC and ultrasound scans were performed in all patients, with a CT requested when necessary to evaluate extrathyroidal tumor invasion.

**Fig. 1:**
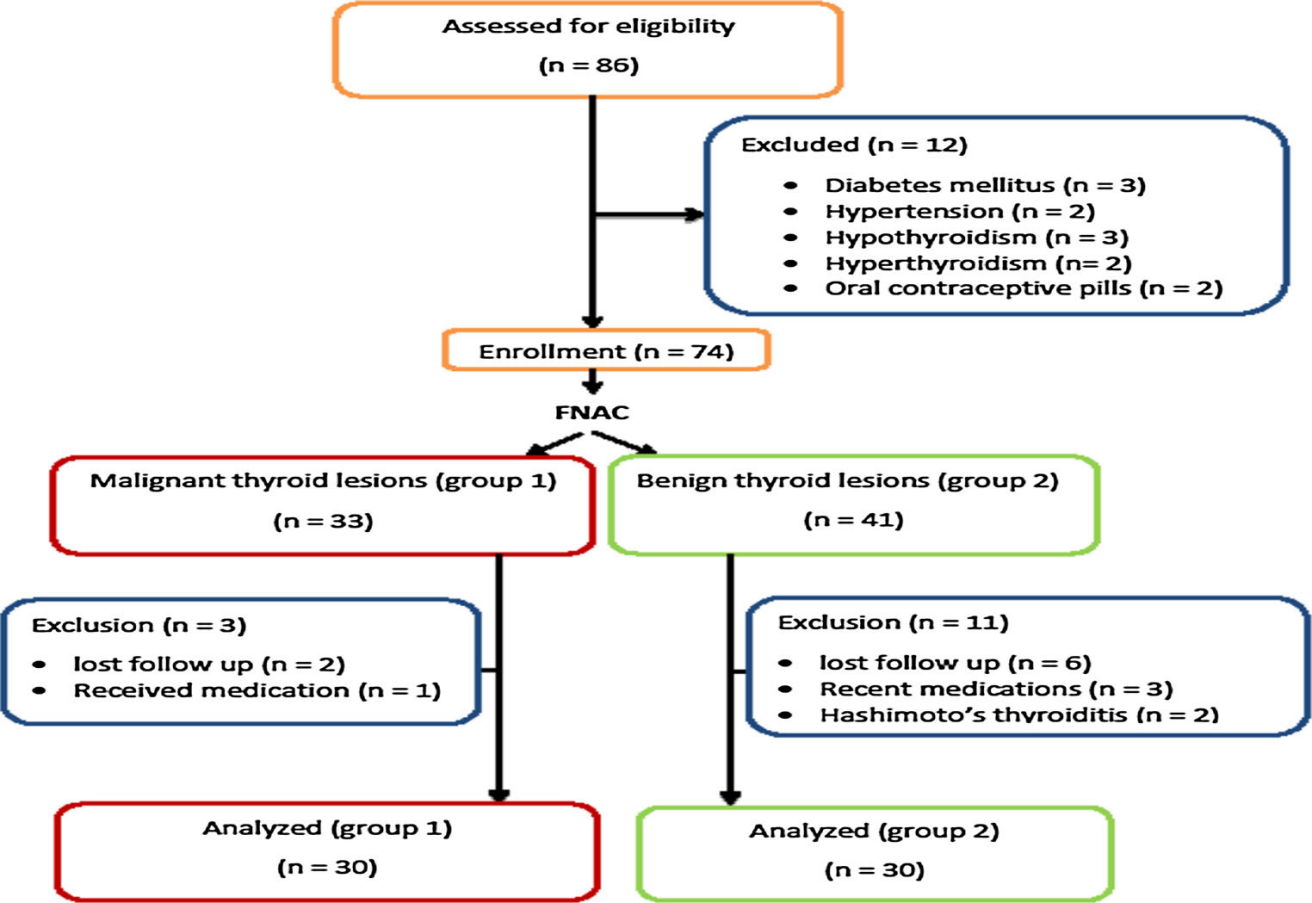
Flow chart showing the patients with thyroid disease excluded from the study

Exclusion criteria included diabetes mellitus, hypertension, thyroid hormonal profile abnormalities, either hyperthyroidism or hypothyroidism, and ongoing hormonal therapy, such as oral contraceptive pills, estrogen preparations, and thyroid hormone preparations. Patients with evidence of recent systemic illness or receiving medications for other medical conditions were also excluded from the study (Fig. 1).

All patients underwent either total thyroidectomy (TT) or hemithyroidectomy. Central neck dissection (CND) was done for those with positive central lymph nodes, and functional neck dissection (FND) was performed in patients with lateral nodal neck disease. Histopathological examination of the resected specimens was performed. Pathological criteria including tumor size, tumor pathological type, extrathyroidal invasion, multifocality, lymphovascular invasion, lymph node status, and presence of lymphocytic thyroiditis were reported.

Patient demographics and anthropometric measurements including weight, height, and body mass index (BMI) were recorded. Samples were collected with the patient in the fasting state in the morning. Sample collection, processing, and storage were done according to the instructions of the reference laboratory and the kits. Samples for leptin level measurements were taken from the patients preoperatively and again 1 month postoperatively.

### Assays

Serum samples were kept at −40 °C until analysis. All samples from each patient were run in the same assay. Serum leptin was measured by the DIAsource Leptin-EASIA Kit, (DIAsource ImmunoAssays, Louvain-la-Neuve, Belgium).

### Statistical analysis

Data were collected, tabulated, and then analyzed using IBM SPSS software package version 20.0., and statistically analyzed with the Shapiro–Wilk test, the D’Agastino test, and the Mann–Whitney test. Significance of the obtained results was judged at the 5 % level.

## Results

In group 1, 15 (50 %) patients underwent TT with FND, 5 (16.7 %) patients underwent TT with CND, 8 (26.7 %) patients underwent TT, and 2 (6.6 %) patients who had a diagnosis of micropapillary carcinoma underwent hemithyroidectomy with no further surgery. In group 2 (controls) 22 (73.3 %) patients underwent TT and 8 (26.7 %) patients underwent hemithyroidectomy. All patients had their leptin levels measured preoperatively and 1 month after the operation.

### Demographics and patient characteristics

There was no significant difference between the two groups regarding age and gender. The BMI for all patients was recorded and compared in both study groups with no significant statistical difference The menstrual status of female patients in both groups was compared with no significance between studied groups (*p* = 0.462) (Table 1).

**Table 1:** Relationship between demographic data and leptin expression

	Group 1	Group 2	*p* value
Gender			
Male	12 (40.0 %)	6 (20.0 %)	0.091
Female	18 (60.0 %)	24 (80.0 %)	
Age, years	55.50 (18.0–67.0)	35.50 (22.0–67.0)	0.157
BMI	24.87 ± 5.84	25.03 ± 4.51	0.902
Menstrual status	*n* = 18	*n* = 24	
Pre-menopause	13 (72.2 %)	20 (83.3 %)	0.462
Post-menopause	5 (27.8 %)	4 (16.7 %)	

### Comparison between serum leptin levels in both study groups

Both preoperative and postoperative serum leptin levels were measured in the two study groups. The preoperative serum leptin levels were significantly higher in the WDTC patients than in the controls (19.25 [1.50–109.60] vs 0.90 [0.50–11.80] ng/ml; *p* < 0.001, group 1 vs group 2, respectively) (Table 2).

**Table 2:** Comparison between cases and controls regarding leptin level

	Group 1	Group 2	*p* value
Preoperative leptin level, ng/ml	19.25 (1.50–109.60)	0.90 (0.50–11.80)	<0.001*
Postoperative leptin level, ng/ml	0.90 (0.60–8.90)	0.80 (0.50–10.80)	0.532
*p* value	<0.001*	0.274	

* Statistically significant

There was a significant drop in serum leptin levels 1 month after surgery in the WDTC group, falling from 19.25 (1.50–109.60) to 0.90 (0.60–8.90) ng/ml (*p* < 0.001). In contrast, for the control group there was no significant difference between preoperative and postoperative serum leptin levels (*p* = 0.274).

One month after surgery, there was no significant difference between serum leptin levels in both groups: 0.90 (0.60–8.90) vs 0.80 (0.50–10.80) ng/ml, group 1 vs group 2 respectively (*p* = 0.532).

### Comparison between leptin levels in different BMI subgroups

A subanalysis of the two groups was performed based on the patients’ BMI. They were subdivided into underweight (BMI <18), normal (BMI 18 to <25), overweight (BMI 25 to <30), and obese (BMI ≥30) (Table 3). Leptin levels were measured and compared in the four subgroups before and after surgery. There was no significant difference between the two groups regarding BMI. There was, however, a significant postoperative decrease in serum leptin levels in all BMI subgroups in WDTC patients when compared to their preoperative serum leptin levels (Table 4).

**Table 3:** Comparison between both groups regarding BMI

	Group 1	Group 2	*p* value
BMI			
Underweight (<18)	5 (16.7)	1 (3.3)	0.195
Normal (18 to <25)	11 (36.7)	13 (43.3)	0.598
Overweight (25 to <30)	7 (23.3)	11 (36.7)	0.260
Obese (≥30)	7 (23.3)	5 (16.7)	0.519
*p* value	0.256

**Table 4:** Comparison between preoperative and postoperative leptin levels in WDTC with respect to BMI subgroups

	Underweight (<18) (*n* = 5)	Normal (18 to <25) (*n* = 11)	Overweight (25 to <30) (*n* = 7)	Obese (≥30) (*n* = 7)
Preoperative leptin level, ng/ml	24.0 (11.50–24.0)	4.20 (1.50–109.60)	22.80 (15.70–57.0)	34.90 (10.0–44.80)
Postoperative leptin level, ng/ml	0.70 (0.70–3.70)	0.80 (0.60–2.10)	0.80 (0.60–4.50)	6.90 (6.80–8.90)
*p* value	0.038*	0.003*	0.017*	0.017*

* Statistically significant

### Comparison between leptin levels according to menopausal status

Of 30 patients in the WDTC group, 18 (60 %) were females, and in the control group there were 24 (80 %) females. Female patients in both groups were classified according to their menstrual status into premenopausal or postmenopausal (Table 5). There was no statistically significant difference between preoperative serum leptin levels in premenopausal and postmenopausal WDTC female patients: 11.50 (1.50–44.80) and 34.90 (10.0–34.90) ng/ml, respectively (*p* = 0.213).

**Table 5:** Comparison between preoperative and postoperative leptin levels in different groups according to menstrual status

	Pre-menopause	Post-menopause	*p* value
Group 1			
*n*	13	5	
Preoperative leptin	11.50 (1.50–44.80)	34.90 (10.0–34.90)	0.213
Postoperative leptin	2.10 (0.60–6.80)	8.90 (6.90–8.90)	0.001*
*p* value	0.001*	0.038*	
Group 2			
*n*	20	4	
Preoperative leptin	2.55 (0.50–11.80)	3.55 (0.60–7.70)	0.640
Postoperative leptin	0.80 (0.50–10.80)	3.20 (0.70–6.50)	0.784
*p* value	0.115	1.000	

* Statistically significant

There was a significant decrease in serum leptin levels one month postoperatively for both premenopausal and postmenopausal WDTC patients (*p* = 0.001 and 0.038), respectively; however, the postmenopausal group of patients still had significantly higher serum leptin levels 8.90 (6.90–8.90) ng/ml in comparison to the premenopausal group 2.10 (0.60–6.80) ng/ml (*p* = 0.001).

In the control group there was no significant difference in serum leptin levels between pre- and postmenopausal female patients either preoperatively or postoperatively (*p* = 0.640 and 0.784), respectively, and there was no significant drop in serum leptin levels after surgery in both pre- and postmenopausal patients groups (*p* = 0.115 and 1.0).

### Relationship between clinicopathological criteria and leptin expression

**Fig. 2:**
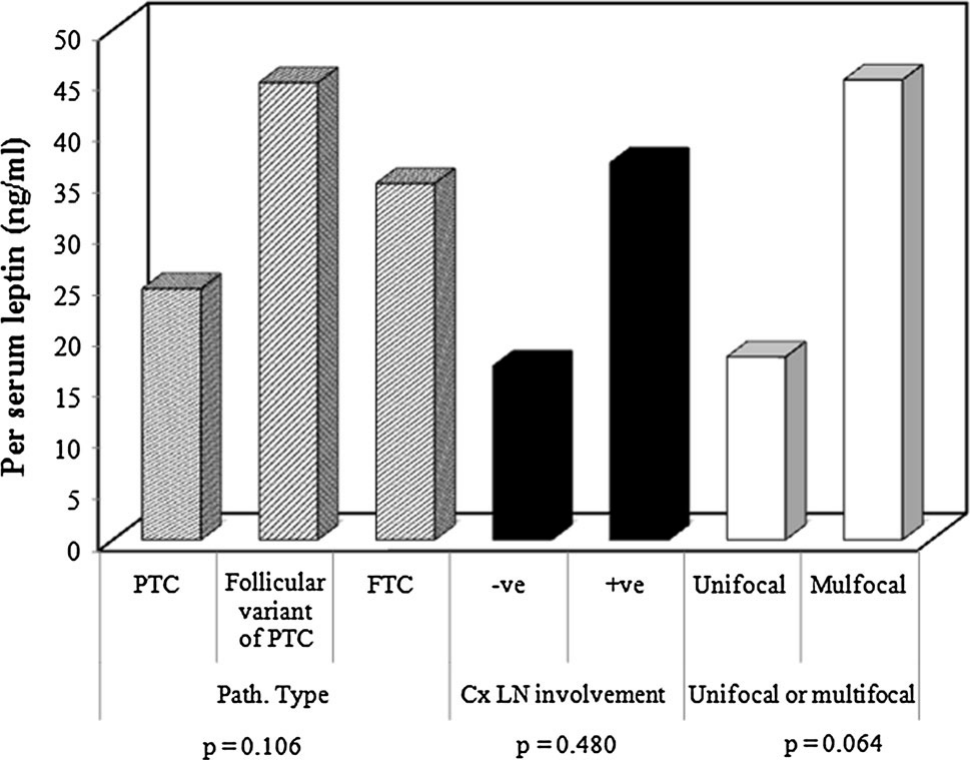
A correlation between histolopathological characteristics and leptin levels. There was no correlation between serum leptin levels and tumor type (*p* = 0.106), cervical lymph node metastases (*p* = 0.48) or number of foci within the thyroid gland (*p* = 0.064), although trends were observed

The postoperative histological examination of specimens from patients with a diagnosis of WDTC showed that 25 (83 %) patients had PTC, 2 (7 %) patients had FVPTC, and 3 (10 %) patients had follicular carcinoma. The comparison of leptin levels of these three groups showed no significant difference (*p* = 0.106), as shown in Fig. 2.

**Fig. 3:**
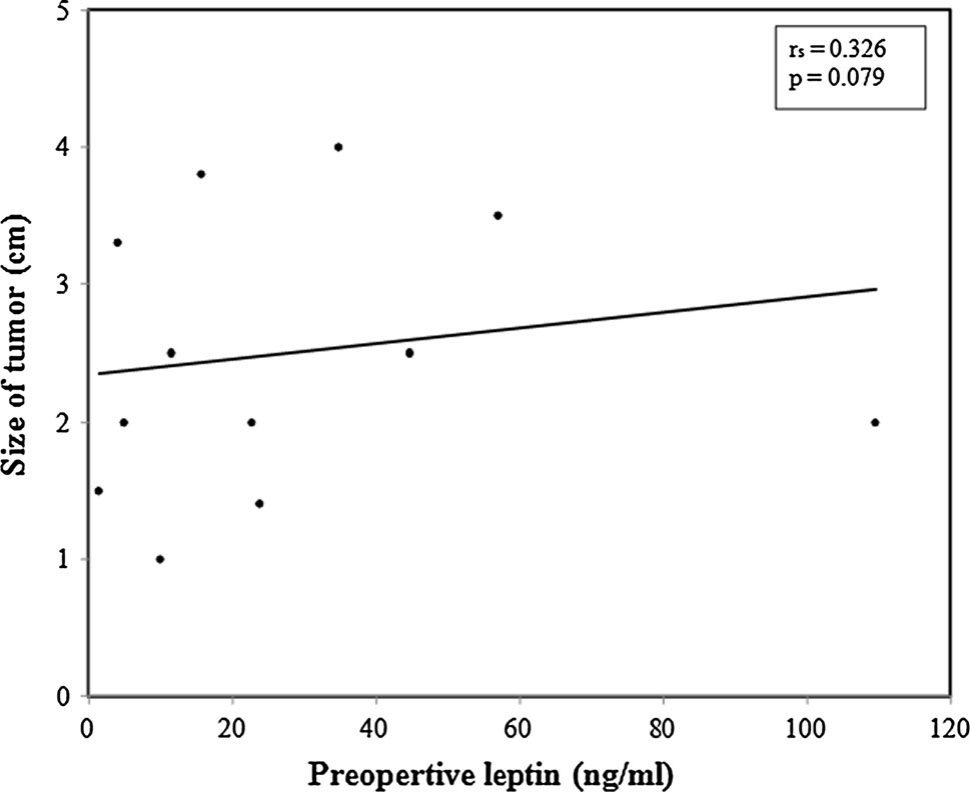
A correlation between primary tumour size and leptin levels. No correlation was observed between the primary cancer size within the thyroid gland and leptin levels (*p* = 0.079)

Lymph node metastases were detected in 20 (66.6 %) patients in the WDTC group. There was a trend for preoperative serum leptin levels to be higher in patients with cervical lymph node metastasis—34.25 (1.50–109.60) ng/ml—compared to those without lymph node metastasis—19.25 (4.20–44.80) ng/ml, however did not reach statistical significance (*p* = 0.480). There was also a trend for serum leptin levels to be higher in multifocal primary thyroid tumors (*p* = 0.064; Fig. 2). A positive trend was also apparent between the size of the tumor and preoperative leptin level; however, it did not reach a significant value (*p* = 0.079; Fig. 3).

## Discussion

Several studies have shown that leptin can act as a growth factor promoting proliferation of transformed cells and stimulating angiogenesis in cancers like endometrium and breast [15, 16]. There is a proven relationship between thyroid gland function and leptin, as both leptin and thyroid hormones regulate the metabolic machinery, as well as energy storage and expenditure. It has been shown that leptin serum levels fluctuate in response to alterations in thyroid activity [17]. This correlation between leptin and thyroid hormone status has been extensively studied, with discrepancies in published results [18–21]. An explanation for these controversial results may arise from the difficulty in matching patients to controls regarding the metabolic status, as the BMI, which is the most commonly used confounding factor is not an accurate reflection of body composition.

Owing to the relation between leptin and both cancer development and the thyroid gland, we hypothesized whether leptin levels would correlate with a diagnosis of thyroid cancer and whether or not higher levels would predict a more aggressive tumor behavior with a greater potential to invade and metastasize. This would reflect on the strictness and duration of patient follow-up and would highlight possibilities of future disease recurrence. This is especially important in Egypt with limited follow-up facilities due to the rural nature, resulting in patients being lost to follow-up and re-presenting several years later with very aggressive disease, as previously reported by our group [22]. In the present study we compared changes in serum leptin levels in patients with WDTC before and after surgery and in control subjects.

Two factors in our study support the relationship between leptin and thyroid cancer. First, we found that the serum leptin levels in WDTC patients were higher than those in their control group counterparts (*p* < 0.001). Second, there was a significant drop in leptin levels 1 month after thyroidectomy compared to pre-thyroidectomy levels. It has been reported that in states of thyroid hormone deficiency, leptin levels increase in parallel with overexpression of thyrotropin releasing hormone and thyroid stimulating hormone (TSH) [17]. It would have therefore been acceptable if leptin levels had increased post-thyroidectomy as a result of the occurrence of intentional hypothyroidism, as we do not replace our thyroid cancer patients with thyroid hormones prior to radioactive iodine ablation. The serum leptin levels did not rise, and the significant decrease in the leptin levels in WDTC patients were paradoxical, and therefore support the concept of a relationship between leptin and thyroid cancer, making leptin a possible etiologic factor in thyroid carcinogenesis. In the benign disease group, there was no significant change in serum leptin levels, possibly due to the fact that these patients were immediately replaced with thyroxine and were euthyroid at the time of leptin measurement.

Following documentation of the primary hypothesis, we sought to investigate other factors that might have influenced leptin serum levels in either group. Because leptin is synthesized and secreted mainly by adipose tissue, and because its plasma levels strongly correlate with BMI [23], we calculated the patients’ BMI in both groups before and after surgery; we could not, however, detect any significant differences. Moreover, leptin levels decreased postoperatively in all subgroups of WDTC patients irrespective of BMI.

Because leptin may also be affected by menopausal status due to associated hormonal changes, both groups were analyzed based on their menopausal status. Although no differences could be detected in either group preoperatively, both premenopausal and postmenopausal women in the WDTC group had a significant reduction in leptin levels following surgery. The postoperative leptin levels in WDTC postmenopausal women, however, remained significantly higher when compared to premenopausal women. This finding contradicts previous studies that reported a decline in serum leptin levels with advancing age and in postmenopausal women [24, 25]. The lack of difference in leptin levels between premenopausal and postmenopausal women in the WDTC group might have been masked by the presence of the cancer as documented earlier. This would have been expected to drop following surgery, especially in the postmenopausal subgroup. The significantly higher leptin levels in postmenopausal women following thyroid surgery could be explained by the hypothyroid status itself, as this did not occur in the benign disease group patients, who were euthyroid. Teixeira et al. [26] reported that in postmenopausal women, there is a stronger influence of thyroid status on leptin levels through a stronger correlation between TSH and leptin leading to higher leptin levels in this population. They showed that this correlation did not occur in premenopausal women; moreover, leptin levels were reduced with thyroxine replacement.

We then investigated the presence of a correlation between histopathological findings and serum leptin levels. In WDTC, the size of the primary tumor, multifocality, and lymph node status are essential criteria in risk stratification into either low risk or high risk and will predict disease recurrence [27, 28]. Although trends were demonstrated between leptin levels and each of tumor size (*p* = 0.079), malignant lymph node involvement (*p* = 0.48), and multifocal disease (*p* = 0.064), the differences did not reach statistical significance. Cheng et al. [29, 30] observed a strong association between leptin tissue expression and both tumor size and metastatic lymph nodes in their clinicopathological study, with further work showing the role of leptin in tumor cell migration in PTC.

To conclude, higher leptin levels were associated with a diagnosis of WDTC with a significant drop in the leptin levels following surgery. Other prognostic factors, like lymph node status, age, tumor size, and multifocal disease, had no effect on leptin levels. Future studies should focus on finding a correlation between cancer activation pathways, leptin receptor tissue expression, and serum leptin levels, which could then be used as a tumor marker in the clinical setting.
